# Metabolic engineering of *Rhodococcus ruber* Chol-4: A cell factory for testosterone production

**DOI:** 10.1371/journal.pone.0220492

**Published:** 2019-07-26

**Authors:** Govinda Guevara, Yamileth Olortegui Flores, Laura Fernández de las Heras, Julián Perera, Juana María Navarro Llorens

**Affiliations:** Department of Biochemistry and Molecular Biology, Facultad de CC, Biológicas, C/Jose Antonio Novais, Universidad Complutense de Madrid, Madrid, Spain; University of California San Diego, UNITED STATES

## Abstract

*Rhodococcus ruber* Chol-4 is a potent steroid degrader that has a great potential as a biotechnological tool. As proof of concept, this work presents testosterone production from 4-androstene-3,17-dione by tailoring innate catabolic enzymes of the steroid catabolism inside the strain. A *R*. *ruber* quadruple mutant was constructed in order to avoid the breakage of the steroid nucleus. At the same time, an inducible expression vector for this strain was developed. The 17-ketoreductase gene from the fungus *Cochliobolus lunatus* was cloned and overexpressed in this vector. The engineered strain was able to produce testosterone from 4-androstene-3,17-dione using glucose for cofactor regeneration with a molar conversion of 61%. It is important to note that 91% of the testosterone was secreted outside the cell after 3 days of cell biotransformation. The results support the idea that *Rhodococcus ruber* Chol-4 can be metabolically engineered and can be used for the production of steroid intermediates.

## Introduction

Steroids are a family of terpenoid lipids widely distributed in nature that present a common structure formed by four fused alicyclic rings called gonane (S1 Fig). They play important biological roles in eukaryotic cell membrane stabilization (e.g. structural steroids such as cholesterol) and the regulation of cellular processes such as proliferation and differentiation (e.g. steroid hormones such as progesterone or testosterone, TS).

Steroid-based drugs are widely used as anti-inflammatory, diuretic, anabolic, contraceptive, anti-androgenic, progestational and anticancer agents and they are therefore a highly valuable resource for the pharmaceutical industry with an annual global market of over $10 billon [1]. Steroids can be chemically produced in a variety of ways, but the most common initial substrates for their production are cost-effective phytosterols, derived from plants [2]. However, in the recent years, there has been an increasing effort to move from the multistep chemical process to a single step microbial bioconversion process that could reduce costs and production yield losses, and would also be more eco- friendly [1]. All this led to an increasing interest in the use of recombinant bacteria as cell biofactories. These compounds are synthesized and found predominantly in eukaryotic cells while some bacteria, that rarely generate them, are able to use these compounds as growth substrates [3].

Currently, this objective is much more achievable thanks to recent advances in our understanding of steroid bacterial catabolism, which led to the development of bacterial strains that can convert precursors (e.g. phytosterols from cheap agricultural plant waste) into high-value steroids [4, 5]. Therefore, in this work, we present *Rhodococcus* as a promising candidate for steroid production.

The cholesterol-degrading *R*. *ruber* strain Chol-4, isolated from a sewage sludge sample, was chosen [6] as a model organism in this work for the following reasons: i) *R*. *ruber* strain Chol-4 is able to catabolize steroids; ii) many of its enzymes involved in the steroid catabolism have been characterized in the past years and iii) an important mutant strain collection listed in S1 Table is now available [7–10]. However, so far, there are only a few studies on the use of engineered *Rhodococcus* as a cell system for pharmaceutical steroid production [11–13].

The proposed catabolic oxidative pathway of cholesterol and phytosterols in many actinobacteria is formed by a complex set of enzymatic reactions that includes an upper pathway (the oxidation to 4-cholesten-3-one and the carbon side chain cleavage at C17, similar to the β-oxidation of fatty acids) while attempting polycyclic ring opening [14–18]. Two key enzymes initiate the opening of the steroid ring: the 3-ketosteroid-Δ^1^-dehydrogenase [4-ene-3-oxosteroid: (acceptor)-1-ene-oxoreductase; EC 1.3.99.4], also known as KstD and the 3-ketosteroid 9α-hydroxylase [Androsta-1,4-diene-3,17-dione; EC 1.14.13.142], known as Ksh [19]. KstD is a flavoenzyme involved in the Δ^1^-dehydrogenation of the steroid molecule leading to the initiation of the breakdown of the steroid nucleus by introducing a double bond into the A-ring (see S1 Fig) of 3-ketosteroids [20, 21]. This flavoprotein converts 4-ene-3-oxosteroids (e.g. 4-androstene-3,17-dione or AD) to 1,4-diene-3-oxosteroids (e.g. 1,4-androstadiene-3,17-dione or ADD) by trans-axial elimination of the C-1(α) and C-2(β) hydrogen atoms [22]. KshAB is an enzymatic complex responsible for C9α-hydroxylation; it consists of a terminal oxygenase (KshA subunit) which performs substrate hydroxylation and a ferredoxin reductase (KshB subunit) which mediates the electron transfer [23].

**Fig 1:**
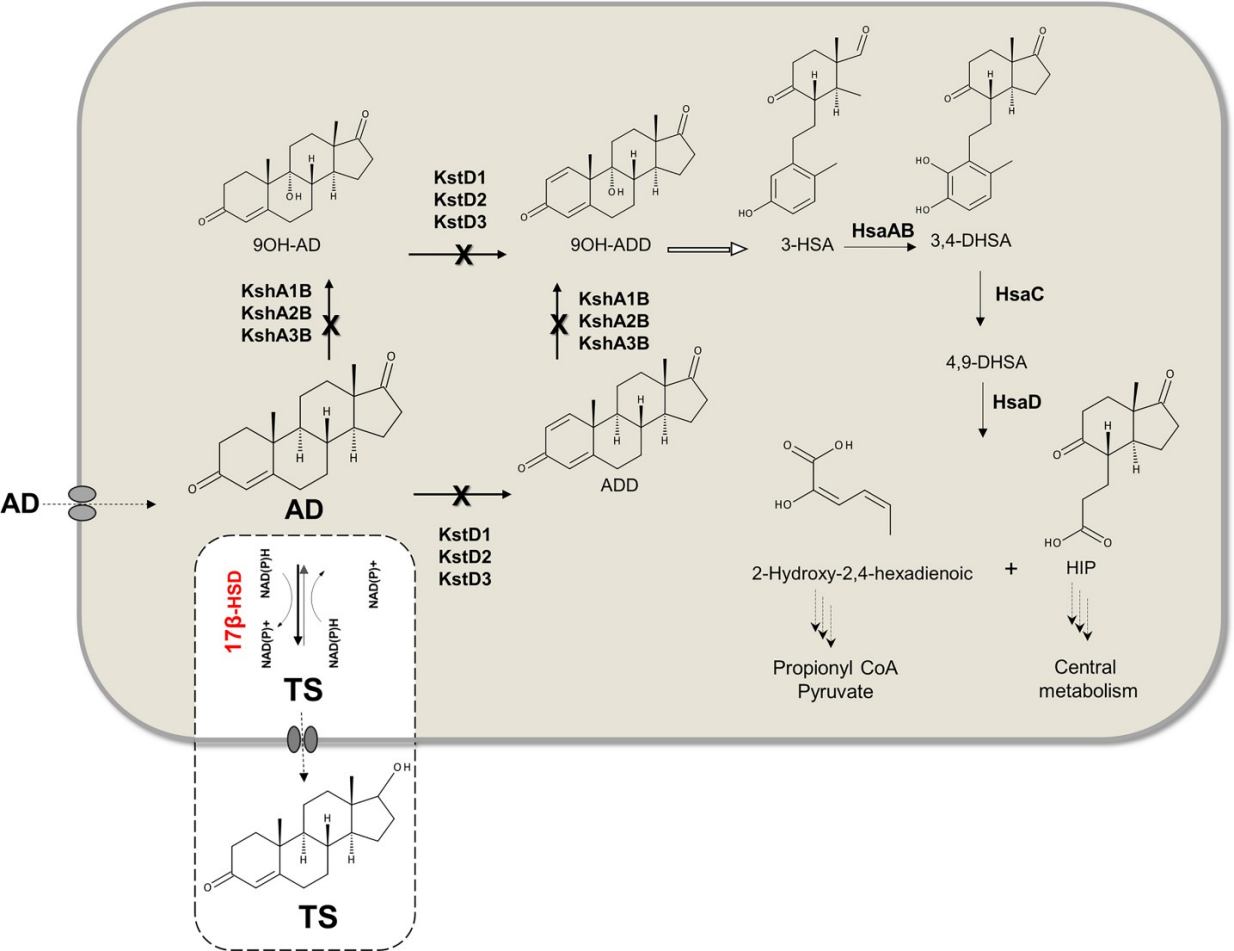
Metabolic network for the production of TS from AD. *Rhodococcus rube*r catabolizes the AD through different steps. The *ΔkshB-kstD*1,2,3 quadruple mutant strain (cross marks point out the mutations done) has the AD catabolic enzymes blocked and therefore it could be used as chassis for the production of AD-derivatives. As a proof of concept, the recombinant strain harboring the 17β-HSD fungal enzyme yielded testosterone from AD. Abbreviations: **AD**: 4-androstene-3,17-dione; **ADD**: 1,4-androstadiene-3,17-dione **TS**: testosterone; **9OH-AD**: 9 alpha-hydroxyandrosta- 1,4-ene-3,17-dione; **9OH-ADD**: 9 alpha-hydroxyandrosta-1,4-diene-3,17-dione; **3-HSA**: 3-hydroxy-9,10-secoandrosta-1,3,5(10)-triene-9,17-dione; **3,4-DHSA**: 3,4-dihydroxy-9,10-secoandrosta-1,3,5(10)-triene-9,17-dione; **4,9-DHSA**: 3-hydroxy-5,9,17-trioxo-4,5:9,10-disecoandrosta-1(10),2-dien-4-oate; **HIP**: 9,17-dioxo-1,2,3,4,10, 19-hexanorandrostan-5-oic acid. **KstD**: 3-ketosteroid Δ1-dehydrogenase; **KshAB**: 3-cetosteroide-9α-hidroxilasa; **HsaAB**: 3-hydroxy-9,10-secoandrosta-1,3,5(10)-triene-9,17-dione monooxygenase; **HsaC**: extradiol dioxygenase; **HsaD**: 9,10-diseco-3-hydroxy-5,9,17-trioxoandrosta-1(10),2-diene-4-oate hydrolase.

AD is a substrate for both KstD and KshAB enzymes that yield ADD and 9α-hydroxy-4-androstene-3,17-dione (9OH-AD), respectively. These products are further transformed with the same combination of enzymes to finally yield the unstable compound 9α-hydroxy-1,4-androstadiene-3,17-dione (9OH-ADD) that spontaneously breaks the B ring to generate 3-hydroxy-9,10-secoandrost-1,3,5(10)-triene-9,17-dione (3-HSA). Finally, the lower catabolic pathway leads to formation of primary metabolites (Fig 1) [15]. When Ksh activity is inactivated, ADD is accumulated as the result of KstD activity on the intermediates generated from side chain degradation. Similarly, 9OH-AD is accumulated after inactivating the KstD activity [15, 24].

A mutant with impaired Ksh and KstD activities is not able to degrade AD, favoring its modification into any molecule of interest with the appropriate enzymatic activity. *Rhodococcus*, just like other actinobacteria, has many isoforms of these enzymes. Fortunately, all *R*. *ruber* KshA isoforms (KshA1, KshA2, KshA3) combine with only one KshB, therefore Ksh activity is absent by knocking down the *kshB* gene [9]. *R*. *ruber* displays three KstD isoforms (KstD1,2,3) and therefore it is necessary to knock down all of them to impair KstD activity [8, 10].

To study the biotechnological capabilities of a *R*. *ruber* system, a biotransformation of AD to testosterone, a natural steroid hormone from the androgen group, was conducted using the fungal enzyme 17-ketosteroid reductase. As the degradation pathway of AD is well characterized in *Rhodococcus ruber*, this substrate was chosen for the study. Assays were not considered on other steroids such as cholesterol or phytosterols as initial substrates because *R*. *ruber* displays more than one steroid pathway, whose major steps remain unknown and do not consequently yield AD as intermediate in this strain [8, 9].

The enzyme 17-ketosteroid reductase (17β-HSD; 17β-hydroxysteroid:NADP 17-oxidoreductase, EC 1.1.1.64) chosen for this work was obtained from the filamentous fungus *Cochliobolus lunatus* (17β-HSDcl) and its DNA sequence was previously codon optimized for the actinobacteria *Mycobacterium* [5]. This enzyme has been subjected to extensive biochemical, kinetic and quantitative structure-activity relationship studies [25–32]. It catalyzes a reversible NAD(P)H/NAD(P)^+^-dependent reduction/oxidation reaction in the hydroxyl/keto groups at the C-17 position of different steroids [33–35], although it is more active in reduction [27, 29–31]. Therefore, 17β-HSDcl was a strong candidate to be used in bacteria to obtain testosterone from AD. Recently, a biological model system for industrial production of testosterone using the enzyme 17β-HSDcl in an engineered *Mycobacterium smegmatis* has been described [5]. A scheme of the transformation process by this enzyme using the *R*. *ruber* mutant is shown in Fig 1. It is important to note that the blocking of Ksh or KstD activities in this strain also yielded a lack of growth on testosterone [9, 10].

To sum up, this work presents *R*. *ruber* as a candidate for steroid biotransformation and, to test the system, testosterone was produced. To achieve this aim, several steps were taken: first, a Chol-4 quadruple mutant Δ*kshB-kstD1*,*2*,*3* strain had to be generated to have a convenient enzymatic cellular background to completely block the AD catabolism (Fig 1); secondly, as no available plasmid was functional on the *R*. *ruber* strain Chol-4, a specific expression vector had to be built for this strain. Finally, it was necessary to obtain a recombinant strain overexpressing the *17β-hsd* gene.

## Results and discussion

### Construction of *ΔkshB-kstD1*,*2*,*3 R*. *ruber* strain Chol-4 mutant

AD is a substrate of both KstD and KshAB activities in *R*. *ruber*. The KstD1,2,3 *R*. *ruber* mutant accumulates 9OH-ADD from AD while the KshB *R*. *ruber* mutant accumulates ADD [8, 9]. Therefore, the first step to obtain steroid derivatives from AD in *R*. *ruber*, is to block its AD catabolic pathway by building a mutant strain in which the activity of these enzymes is impaired (Fig 1). Besides avoiding the AD consumption, the quadruple mutant Δ*kshB-kstD1*,*2*,*3* has also blocked the testosterone catabolism.

In order to get the quadruple mutant, the *ΔkstD1*,*2*,*3* strain [8] (S1 Table) was used for *kshB* gene deletion, according to Material and methods section. The *ΔkshB-kstD1*,*2*,*3* quadruple mutant was checked by PCR. The growth experiments proved that it was not able to grow on 2 mM AD, ADD or testosterone while it kept growing on 24 mM sodium acetate (S2 Fig). The slight growth seen on cholesterol is due to the consumption of the side chain of this substrate [9]. Therefore, the quadruple mutant was suitable to be used as a host for the *17β-hsd* overexpression for AD biotransformation.

### Construction of an expression vector for *R*. *ruber* Chol-4 and *17β-hsd* gene cloning

Attempts to transform *R*. *ruber* Chol-4 with expression vectors (pTIP-QC1, pNIT-QC1, pTNR-KA, pTNR-TA, etc.) were unsuccessful. The biotechnological potential use of *R*. *ruber* was restricted by the need for an appropriate expression vector.

**Fig 2:**
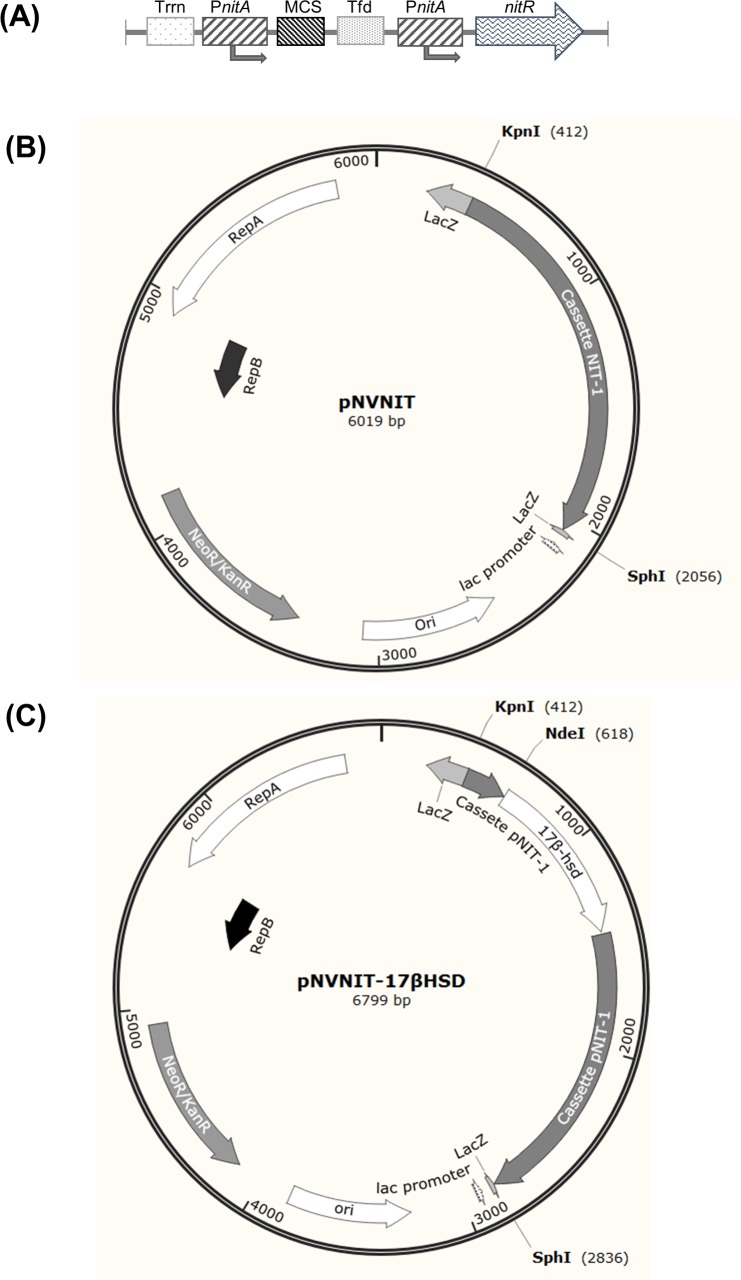
Construction of pNVNIT-17βHSD vector. (A) Scheme of the NIT-1 cassette adapted from Pandey *et al*. 2009 [37]. P*nitA*: promoter of the *nitR* gene; NitR: regulatory protein; MCS: multiple cloning site. Trrn and Tfd stand for two strong terminator sequences, one derived from the *E*. *c*oli *rrnAB* operon and the other one from the bacteriophage fd respectively The NIT-1 cassette was cloned in the *Kpn*I-*Sph*I sites of the pNV119 vector to generate the pNVNIT vector. (B) Scheme of the pNVNIT vector. (C) The *C*. *lunatus* 17β*-hsd* gen was cloned in the *NdeI-DraI* sites of the pNVNIT vector yielding to pNVNIT-17βHSD.

To create an inducible expression vector for *R*. *ruber*, we combined the pNV119 vector, which was able to replicate in this strain, and an artificial regulon (GenBank: FjI73069) [36] that contains a gene encoding a regulatory protein (NitR) under the control of the inducible promoter *PnitA* and a separate cistron with a second *PnitA* promoter followed by a multiple cloning site (MCS). The native ribosome binding site of the *nitR* gene was preserved in the MCS. This arrangement of regulatory elements creates a positive-feedback loop in which the expression of both the regulatory protein and the target gene are simultaneously induced (Fig 2A). The inductor is the nitrile derivative ε-caprolactam [36]. This cassette, called NIT-1, was synthesized for this work by Invitrogen. The pNV119 derived vector containing this cassette was called pNVNIT (Fig 2A). *R*. *ruber* was successfully transformed with this vector, providing a way to check its potential as a functional expression vector for this strain.

Similarly to *M*. *smegmatis* mc2155 [5], we were not able to identify any 17β-HSD homologous enzyme in *R*. *ruber* Chol-4 by blasting the protein sequence against its genome (GenBank::NZ_ANGC00000000.2). Therefore, a synthetic variant of the fungal enzyme 17β-HSD codon-optimized for actinobacteria and efficient in the actinobacteria *Mycobacterium* [5] was chosen to be cloned and overexpressed in *R*. *ruber*.

Finally, the synthetic *C*. *lunatus 17β-hsd* gene was obtained from the pUC57-17HSD plasmid [5] and cloned into the pNVNIT vector (Fig 2B and 2C), giving rise to the pNVNIT-17βHSD plasmid.

### Expression of 17β-HSD recombinant protein and AD biotransformation

**Fig 3:**
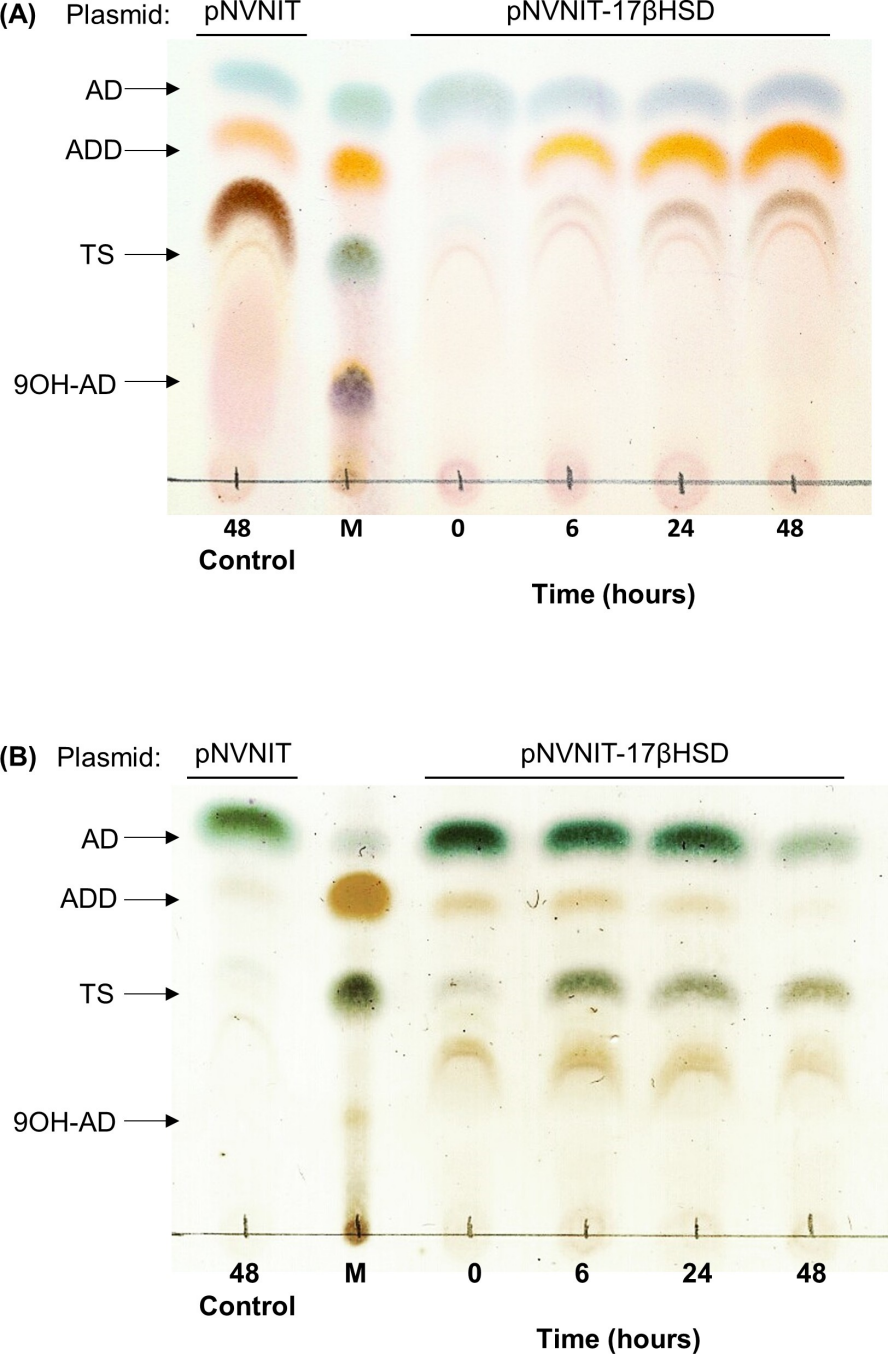
Analysis of Testosterone production from AD by TLC. The strains were grown in LB at 30°C and 250 rpm until a 0.8 OD_600nm_ was reached and induced with 28 mM ε-caprolactam for 24 hours. The biotransformation assay started by adding 1 mg/mL of AD and samples were taken at different times. (A) TLC of wild-type (WT) strain harboring the pNVNIT-17βHSD (line 3 to 6). (B) TLC of the *ΔkshB-kstD1*,*2*,*3* mutant harboring the pNVNIT-17βHSD plasmid (line 3 to 6). The control (line 1) in Figs A) and B) belongs to the strains WT or *ΔkshB-kstD1*,*2*,*3* harboring the empty plasmid pNVNIT at 48 hours after the AD addition. M (line 2): markers AD, ADD, TS (testosterone) and 9OH-AD (1 μg/μL).

The wild-type (WT) and the knockout mutant strain (*ΔkshB-kstD1*,*2*,*3*) were transformed with two plasmids: pNVNIT (as control, Fig 2B) and pNVNIT-17βHSD expression vector (Fig 2C). Recombinants harboring the plasmids were used for a growing-cell biotransformation study. As a first approach to detect testosterone, a culture of each strain (wild-type and mutant strain transformed with both plasmids) were grown on LB. After 16–24 hours (0.8–1.0 DO_600nm_), the ε-caprolactam inductor was added and 24 hours later, 1 mg/mL of AD was added in powder form. Samples were taken at different times for analysis by Thin-layer chromatography (TLC). As it is shown in Fig 3A, line 1, the wild-type strain, harboring the pNVNIT plasmid, progressively converted the AD into ADD, as its metabolic machinery is kept intact. The quadruple *R*. *ruber* mutant, also containing the pNVNIT plasmid, but with the steroid catabolic pathway impaired, was unable to metabolize AD (Fig 3B, line 1).

In the case of the quadruple mutant harboring the pNVNIT-17βHSD vector, the biotransformation of AD to TS can only be seen 6 hours after the addition of AD.

The wild-type strain expressing 17βHSD does not produce any TS detectable by TLC (Fig 3). The WT strain can deploy its complete catabolic system to consume the AD entering into the cell. Therefore, it could be expected that the metabolic flow goes toward AD degradation rather than testosterone production. Moreover, if testosterone was produced, it could soon be degraded by the intact cell machinery and by no means detected by TLC or HPLC.

The TLC analysis of the quadruple mutant also showed two spots: one with the same RF than the ADD standard and another an unknown product.The presence of the last one in the TLC might be a LB medium metabolite since the spot disappears when the biotransformation takes place in minimal media. The product that was expected to be ADD by TLC (Fig 3B) was not found by HPLC, and therefore it is not ADD. The presense of this product could be explained due to the action of other enzymes different to KstD1,2,3 that might function as a ketosteroid dehydrogenase in *R*. *ruber* and that could alternatively yield an additional product.Minimum medium instead of LB medium was used for the rest of the experiments in order to avoid contamination with undesired metabolites.

These results suggest that the pNVNIT can be an excellent expression vector for *R*. *ruber*, a vector that can broaden the use of this bacteria for biotransformation processes.

**Fig 4:**
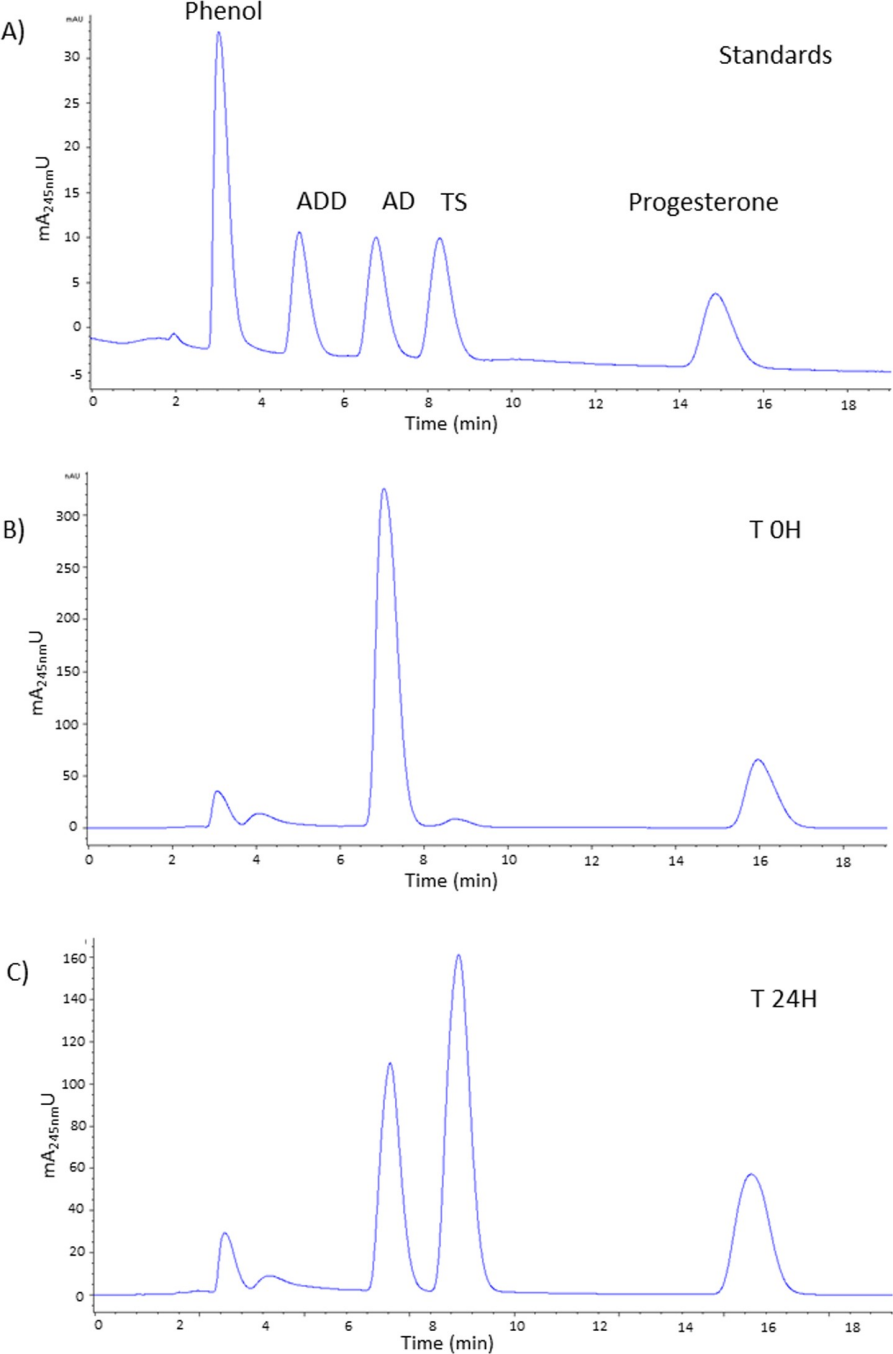
HPLC chromatograms of Testosterone production using culture cells of *R*. *ruber* mutant strain *ΔkshB-kstD1*,*2*,*3*. Cultures harboring the pNVNIT-17βHSD plasmid were incubated at 30°C and 250 rpm in MM until they reached 0.8 OD_600mn_. Heterologous protein expression was induced with ε-caprolactam (28 mM) for 24 h and then AD (1 mM) was added in a Tyloxapol solution (10% v/v). Samples were taken at different times. (A) Standards chromatogram of Phenol (2 mM), ADD, AD, TS and Progesterone (all at 25 μM). (B) Sample took at 0 hours after adding AD and (C) Testosterone production after 24 hours of AD biotransformation. Progesterone was used as an extraction control and phenol as an HPLC internal standard.

The ability to produce TS from AD was verified by HPLC in the recombinant *R*. *ruber* cells supplemented with 1 mM AD (Fig 4). TS production observed was 410 μM 24 hours after the ε-caprolactam induction of the culture with a molar conversion rate of AD to TS of 48.2 ± 3.9%.

On the other hand, the activity of the enzyme 17β-HSD involves a nicotinamide cofactor [26, 37, 38]. The ratio between the oxidized and the reduced form of this type of cofactors plays a crucial role in microbial redox reactions and energy metabolism. The regeneration of this ratio is an important step to be taken into account in biocatalysis [39]. Moreover, *in vivo*, this kind of enzymes displayed a directional preference that depends on the relative affinity for nicotinamide cofactors [NAD(P)(H)] and existing cofactor gradients. For instance, ketosteroid reduction could be favored by keeping the NADPH/NADP^+^ ratio high [37, 38]. Therefore, the cellular ratios of NAD^+^:NADH or NADP^+^:NADPH would encourage the production of TS from steroids rather than the steroid concentration itself [37].

To restore the metabolic cofactor ratio, a supplemental carbon source must be added during the biotransformation studies [38]. Usually, cofactor regeneration is performed using glucose as a co-substrate because of its easy availability and low cost [40] and therefore, the effect of glucose was tested in the recombinant strain of this study.

**Fig 5:**
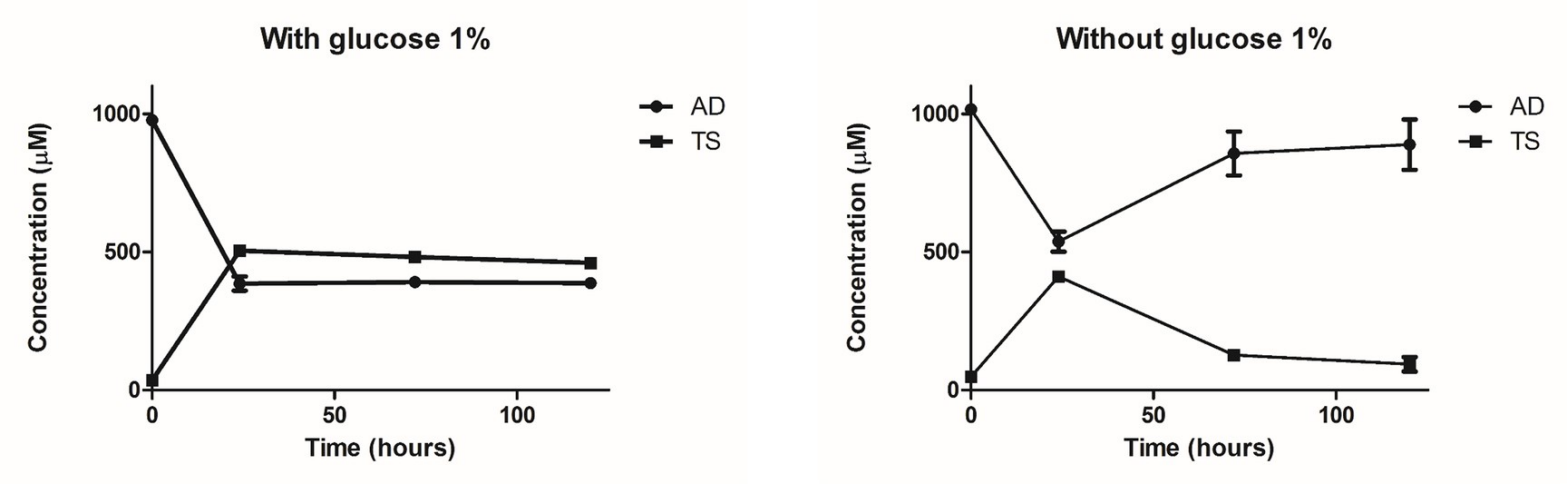
Time course of TS production from AD in the recombinant *R*. *ruber* cells. *ΔkshB-kstD1*,*2*,*3 R*. *ruber* harboring the pNVNIT-17βHSD construction was grown in minimal medium with sodium acetate as carbon source, at 30°C and 250 rpm until 0.8 OD_600nm_ was reached. After 24 hours of the NIT-1 regulon induction (ε-caprolactam at 28 mM) the biotransformation started by adding AD at 1 mM with or without 1% w/v glucose. Samples were taken at different times, extracted by chloroform and analyzed by HPLC. *ΔkshB-kstD1*,*2*,*3 R*. *ruber* harboring the pNVNIT vector as control were also tested and no testosterone was detected (not shown).Testosterone and AD concentration were determined as indicated in Material and methods. Average and standard deviation of three biological replicates are shown.

The time course of TS production from AD was analyzed with and without glucose in the medium (Fig 5). By adding glucose 48h after the inoculation, cell density increased from 2.5 ± 0.3 to 8.6 ± 0.5. This fact raises the molar conversion rate of AD to TS after 24 hours of bioconversion from 48.2 ± 3.9% to 61.5 ± 3.0%. The cell culture grown with glucose in the medium, kept AD conversion constant over time in comparison with the culture grown without glucose (Fig 5).

This result is consistent with the effects of the supplemented carbon source detected in TS production in other strains [41–43]. However, the increase in the molar converstion rate at 24 hours was not as high as the one reported in *Mycobacterium* resting-cells (from around 35% in the absence of glucose to 90% molar conversion rate in a medium supplemented with glucose) [5] reinforcing the idea that this reaction could depend on the specific metabolic context of the cell.

The maximum conversion rate of 61% obtained for the recombinant *R*. *ruber* is consistent with the conversion range from AD to TS described in growing cells which vary from 27% (*Saccharomyces cerevisiae* [44]) to 93% (*Zygowilliopsis* sp. WY7905 [43]). TS productivities in other bacterial cell-factories are shown on S2 Table. Optimization of this process in the recombinant *R*. *ruber* to obtain a better yield will need a more detailed study.

**Fig 6:**
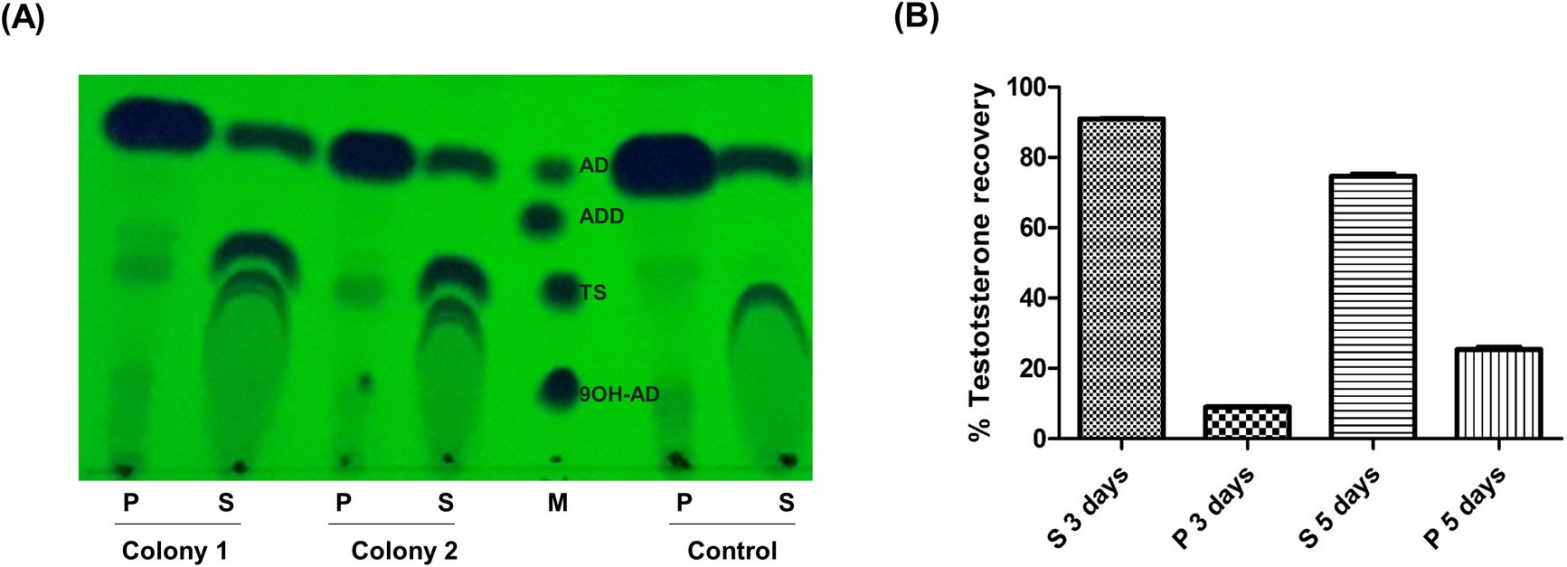
Testosterone distribution in the cell culture. The *ΔkshB-kstD1*,*2*,*3* mutant harboring the pNVNIT-17βHSD plasmid was grown in minimal medium with sodium acetate 24 mM as carboun source at 30°C and 250 rpm. After 24 hours induction, the biotransformation assay started by adding 1 mg/mL of AD. Samples were taken at different times, centrifuged and separated in pellet (P) and supernatant (S), the steroids were extracted accoding to Materials and Methods. (A) TLC of two independent colonies after 3 days of biotransformation. (B) Amount of testosterone detected in P and S at 3 and 5 days of biotransformation measured by HPLC. Control: mutant strain harboring the empty pNVNIT vector. M: markers AD, ADD, TS (testosterone) and 9OH-AD (1 μg/μL).

Lastly, we looked where the testosterone produced by this biotransformation process remained in the cell. After the biotransformation experiment, recombinant *R*. *ruber* cells were centrifuged and pellet and supernatant were separately prepared and analyzed by TLC and HPLC (Fig 6). After 3 days of biotransformation, 91% of the testosterone appeared outside the cell, indicating that the recombinant strain whose testosterone catabolic pathway is blocked due to the lack of KstD and Ksh activities, could get rid of the compound to avoid its intracellular accumulation. After 5 days of biotransformation, this percentage decreases to 75%. The reduction of the testosterone found extracellularly from 91% to 75% is not easy to explain and requires further studies. It could be possible that providing enough time, a putative induction of testosterone-transporter gene expression and an increased re-uptake of testosterone could occur in the cell and in this way favor the entry of TS from the extracellular medium.

The fact that most of the product stayed in the supernatant within the first three days of the assay shows that there is an easy way to recover the TS after a biotransformation process. Some further improvements that need to be optimized may include the rational design of the enzyme itself to obtain a better testosterone yield. Some attempts to modify the fungal enzyme have been made [25] but they have not been tested so far in a biofactory system. Further enzymatic engineering of this activity together with an in-depth study via system biology to optimize the metabolic state of the cell would help to improve the whole production process.

## Conclusions

There is a great interest in the reduction of 17-oxosteroids to 17β-hydroxysteroids as an important way of preparing many steroidal drugs and valuable intermediates. In this study, *R*. *ruber* has been metabolically engineered to effectively convert AD to testosterone as proof of its potential as a cellular factory. Genes involved in steroid ring breakage were knocked out to prevent AD and testosterone catabolism and the 17β-HSD enzyme from *Cochliobolus lunatus* was overexpressed using an expression vector specifically designed for *R*. *ruber*. The recombinant strain produces a yield of 61% testosterone from AD after the biotransformation studies. 91% of the testosterone was recovered extracellularly, broadening the chances of this strain to function as a steroid factory. The whole process still requires optimizing to achieve higher conversion yields, but this work validates the promising use of *R*. *ruber* for the biotechnological production of steroids such as testosterone.

## Material and methods

### Chemicals

AD was kindly given by Gadea Pharmaceutical Group. Chloroform, methanol and phenol of HPLC quality, were supplied by Scharlab S.L. Progesterone, testosterone, sodium acetate, acetone, tyloxapol, antiobiotics, ε-Caprolactam and glucose, were provided by Sigma-Aldrich. Sulphuric acid was obtained from Panreac Química S.A.U.

### Bacterial strains, culture conditions and DNA manipulation

The bacterial strains and plasmids used in this work are listed in Table 1. *Escherichia coli* cells were grown in Luria Bertani (LB) broth in an orbital shaker, at 250 rpm [45] or on LB plates containing the appropriate antibiotics at 37°C. *R*. *ruber* and the mutant strains were routinely grown on LB or minimal medium (Medium 457 of the DSMZ, Braunschweig, Germany) containing the desired carbon and energy source under aerobic conditions at 30°C in a rotary shaker (250 rpm) for 1–3 days. When necessary, antibiotics were added to the medium at 15 μg/mL nalidixic acid and at 50 μg/mL or 200 μg/mL kanamycin for *E*. *coli or R*. *ruber* respectively.

**Table 1:** Bacterial strains and plasmids used in this work.

Bacteria and plasmids	Description	Reference
*Rhodococcus ruber* strain Chol-4	Wild type phenotype, Nal^R^	CECT7469 [6]
*R*. *ruber* Chol-4 *ΔkstD1*,*2*,*3*	*kstD1*, *kstD2* and *kstD3* triple deletion mutant, Nal^R^	[8]
*R*. *ruber* Chol-4 *ΔkshB-kstD1*,*2*,*3*	*kshB*, *kstD1*, *kstD2* and *kstD3* quadruple deletion mutant, Nal^R^	This work
*E*. *coli* DH5α	F’ *endA1 hsdR17* (r_K_^-^m_K_^+^) *glnV44 thi-1 recA1 gyrA*(Nal^r^) *relA1 Δ*(*lacIZYA-argF*) *U169deoR* (ϕ80*dlac*Δ(*lacZ*)*M15*)	Laboratory collection
*E*. *coli* S17-1	*recA pro hsdR RP4-2-Tc*::*Mu-Km*::*Tn7*	[46]
pK18(*ksh*BU+D)	pK18mobsacB harbouring a *Eco*RI-*Pst*I *R*. *ruber* strain Chol-4 genomic fragment containing a *kshB* truncated ORF	[10]
pMK_RQ	Vector harbouring the synthetic cassette NIT-1	Invitrogene
pUC57-17βHSD	pUC57 harbouring the synthetic gene encoding the 17β-*hsd* from *C*. *lunatus*	[5]
pNV119	*KmR*, *Nocardia-E*. *coli* replicative shuttle vector	[47]
pNVNIT	*Rhodococcus*–*Escherichia coli*, expression and shutlle vector, Km^r^	This work
pNVNIT-17HSD	*Rhodococcus*–*Escherichia coli*, expression and shutlle vector harbouring the *17β-hsd* gene, Km^r^	This work

For the biotransformation experiments, an LB pre-grown cultures were washed twice with minimal medium prior to inoculation to 10 mL of fresh minimal medium (initial 0.05 DO_600nm_) supplemented with sodium acetate at 24 mM as the only energy and carbon source.

Competent and electrocompetent cells of *E*. *coli* were prepared and transformed as previously described [45]. Selection of transformed cells was carried out in LB agar plates supplemented with the appropriate antibiotics.

Electroporation of 200 μL of *R*. *ruber* cells was made with 1 μg DNA at 400 Ω, 25 mA, 2.5 μF, 10–11 milliseconds; the resulting cells were suspended in 800 μL of LB and kept for 6 min at 46°C, and then for 5 hours at 30°C without shaking. They were finally plated on LB Agar with 200 μg/mL kanamycin, 15 μg/mL nalidixic acid and kept at 30°C.

The verification of the plasmid transformation of the *R*. *ruber* strains was made in several steps: first, plasmids were extracted from *Rhodococcus*; second, *E*. *coli* was transformed with the plasmid preparation and lastly, *E*. *coli* transformation was checked by standard methods.

All DNA manipulations were performed according to standard molecular cloning procedures [45] or following manufacturers’ instructions (NZYMiniprep and NZYGelpure from NZYtech). DNA sequencing was performed with an ABI Prism 377 automated DNA sequencer (Applied Biosystems Inc.) at Secugen S.L. (Madrid, Spain).

### Construction of the *R*. *ruber ΔkshB-kstD1*,*2*,*3* mutant strain

The *kshB* gene was chromosomally deleted for this work using the *R*. *ruber* triple mutant *ΔkstD1*,*2*,*3* as host and following the unmarked gene deletion method previously described [8] using the plasmid pK18(*ksh*BU+D) [9] and the *E*. *coli* strain S17-1 (Table 1).

The construction of the deleted strain was verified by PCR using the primers CH564 (5’- CGCGTCTCTCCTGATGTGTCGG) and CH331 (5’- ACGTAGCCTGCCTCGATGTCC), at Tm 55°C, 1.5 min and 30 cycles, and further DNA sequencing. In all cases, the Expand High-Fidelity Taq DNA Polymerase from Roche was used for PCR reactions in a Mastercycler personal (Eppendorf). Restriction enzymes and DNA modifying enzymes were purchased to Takara Bio Inc. and New England Biolabs (UK).

### Construction of an expression plasmid for *R*. *ruber* and *17β-hsd* gene cloning

The *Nocardial* high-copy-number cloning vector pNV119 [48] was modified to be used as *a R*. *ruber* expression vector. The NIT-1 cassette (GenBank: FJ173069), an artificial bacterial regulon developed for mycobacteria [36], was synthesized (Invitrogen) and cloned into the *Kpn*I-*Sph*I restriction sites of the pNV119 plasmid. This regulon can use an inexpensive and non-toxic nitrile analog, ε-caprolactam, as an inducer [36]. The resulting plasmid pNVNIT was checked by restriction analysis and sequenced.

On the other hand, the *17β-hsd* gene of *C*. *lunatus* with an optimized codon usage for *Mycbacterium* expression was obtained from digestion of the plasmid pUC57-17HSD [5] with *Nde*I-*Hinc*II restriction enzymes and cloned into the pNVNIT expression plasmid previously digested with *Ned*I-*Dra*I yielding pNVNIT-17βHSD. This plasmid and a control vector without the *17β-hsd* gene were used to electroporate both *R*. *ruber* wild-type and the *ΔkshB-kstD1*,*2*,*3* mutant.

The primers employed to confirm the cloning of the pNIT-1 cassette and *17β-hsd* gene were F24 (5’-CGCCAGGGTTTTCCCAGTCACGAC) and R24 (5’-AGCGGATAACAATTTCACACAGGA), at Tm 58°C, 1 min, 30 cycles.

### Monitoring of AD biotransformation into testosterone by analytical methods

The *ΔkshB-kstD1*,*2*,*3* strain and *R*. *ruber* wild type were electroporated with either the empty plasmid pNVNIT as control or the plasmid harboring the *17*β-*hsd* gen of *C*. *lunatus* (pNVNIT-17βHSD). The recombinant strains were grown in 10 mL of LB medium or minimal medium (MM) with sodium acetate (24 mM) as carbon and energy source with 200 μg/mL kanamycin and 15 μg/mL nalidixic acid, at 30°C in a rotary shaker (250 rpm). Cells were grown up to 0.8 OD_600nm_, then induced with 28 mM ε- caprolactam for 24h; at this point the cultures were in stationary phase with an OD_600nm_ 2.5 ± 0.3 (MM) or OD_600nm_ 9.0 ± 1.0 (LB). Afterwards, 1 mg/mL AD (LB cultures) or 1 mM AD with or without 1% (w/v) glucose (MM cultures) was added. Due to the low solubility of this steroid, the 1 mM AD was prepared from a 10 mM stock dissolved by sonication in 10% (w/v) tyloxapol. The stock solution was autoclaved and kept at room temperature prior to its addition to the minimal medium. Aliquots of 500 μL of the cell culture were taken at fixed times up to 120 hours.

For the analytical studies, the lipid fraction of the samples was obtained by double extraction with 1 mL chloroform and left to dry at 65°C. Two analytical methods were used: the first approach by TLC and a second one by HPLC.

The AD in the TLC experiment was added as powder because the presence of cyclodextrins or tyloxapol damages the TLC plate.

50 μL of chloroform were added to every dried sample and an aliquot of 5 μL was applied onto aluminium TLC Silica gel 60 F254 sheets (Merck). Chromatography was performed using chloroform:acetone (9:1 v/v) as solvent and spots were revealed by UV exposure (λ_254nm_). Afterwards, the TLC plate was dunked into a sulphuric acid:methanol solution (1:9 v/v) followed by a drying step with warm air and heating 1 min at 100°C. 1 μg of standard control samples (testosterone, AD, ADD, and 9OH-AD) were also included in the analysis.

For the reverse-phase HPLC analysis, samples were resuspended in 600 μL chloroform and filtered. Steroids were separated on a Teknokroma mediterranea^TM^ Sea_18_ column (15 cm x 0.46 cm; 5 μM) and UV detected at 245 nm at room temperature. The mobile phase was composed of methanol and water (70/30 v/v) at a flow rate of 1 mL/min. Progesterone was used as an extraction control and phenol as an internal standard on HPLC. AD, ADD, progesterone, and testosterone were used as steroid standards. The conversion rate of TS was calculated on the basis of AD measured into the sample in the resting-cell biotransformations.

## Supporting information

S1 Fig The steroid chemical structure. A) Steroids are a group of natural compounds derived from the hydrophobic and planar gonane nucleus. This carbon backbone core is composed of four rings: three six-member cyclohexane rings (A, B and C) and one five-member cyclopentane ring (D). Steroids vary from one another in the nature of the functional groups attached to the D ring and in the oxidation state. B) One example of steroid is Cholesterol that contains a polar hydroxyl group and a short hydrocarbon tail. The substituents in α configuration are represented by broken lines; substituents in β configuration, with solid lines. Carbon atoms are numbered.
(TIF)

S2 Fig Steroids growth experiments of *R. ruber* wild-type and *ΔkshB-kstD1,2,3* mutant strains. Cultures in minimal media at 30°C and 250 rpm, containing 24 mM sodium acetate, 2 mM AD, 2 mM ADD, 1.8 mM cholesterol or 2 mM testosterone as the only carbon source after 48 hours of growth.
(TIF)

S1 Table List of *R. ruber* steroid mutants available. (DOCX)

S2 Table Microbial conversion of natural sterols to testosterone. (DOCX)

## Abbreviations

AD: 4-androstene-3,17-dione
17β-HSD: 17β-hydroxysteroid oxidoreductase
TS: testosterone
KstD: Ksh3-ketosteroid-Δ^1^-dehydrogenases
Ksh: 3-ketosteroid-9α-hydroxylase
AD: 4-androstene-3,17-dione
ADD: 1,4-androstadiene-3,17-dione
9OH-AD: 9α-hydroxy-4-androstene-3,17-dione
9OH-ADD: 9α-hydroxy-1,4-androstadiene-3,17-dione
HAS: 3-hydroxy-9,10-secoandrost-1,3,5(10)-triene-9,17-dione
TLC: Thin-layer chromatography
