# Supplementary figures and images for: Metabolic engineering of Rhodococcus ruber Chol-4: A cell factory for testosterone production
Source: PLoS One. 2019 Jul 26;14(7):e0220492. doi: 10.1371/journal.pone.0220492 (PMC6660089; doi:10.1371/journal.pone.0220492)

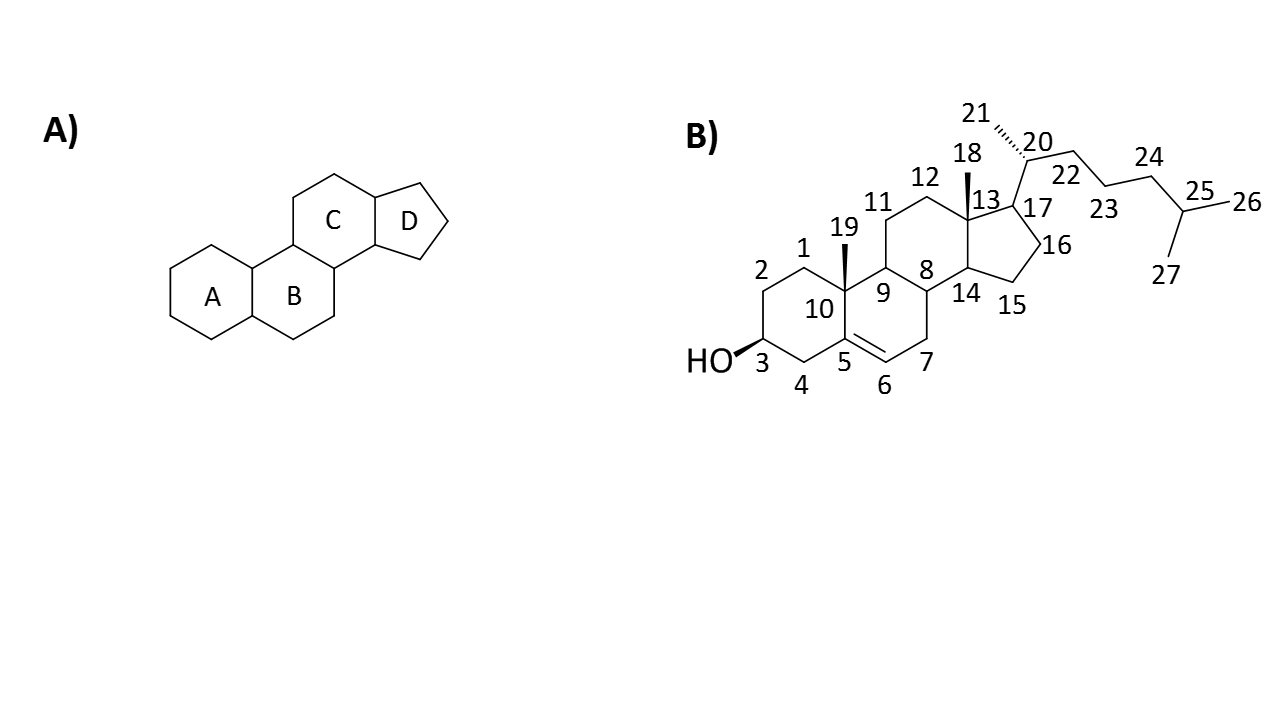

Supplement: S1 Fig — A) Steroids are a group of natural compounds derived from the hydrophobic and planar gonane nucleus. This carbon backbone core is composed of four rings: three six-member cyclohexane rings (A, B and C) and one five-member cyclopentane ring (D). Steroids vary from one another in the nature of the functional groups attached to the D ring and in the oxidation state. B) One example of steroid is Cholesterol that contains a polar hydroxyl group and a short hydrocarbon tail. The substituents in α configuration are represented by broken lines; substituents in β configuration, with solid lines. Carbon atoms are numbered. (TIF) [file pone.0220492.s001.tif]

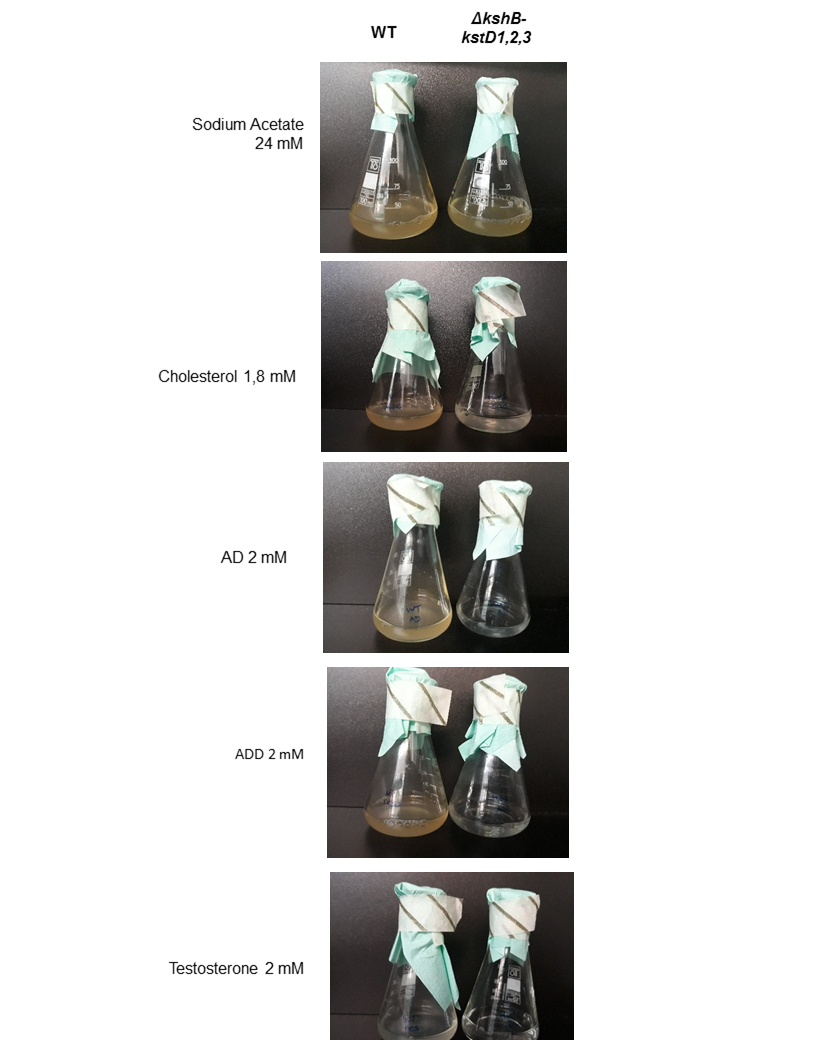

Supplement: S2 Fig — Cultures in minimal media at 30°C and 250 rpm, containing 24 mM sodium acetate, 2 mM AD, 2 mM ADD, 1.8 mM cholesterol or 2 mM testosterone as the only carbon source after 48 hours of growth. (TIF) [file pone.0220492.s002.tif]
