# Supplementary material for: Metabolic engineering of Rhodococcus ruber Chol-4: A cell factory for testosterone production
Source: PLoS One. 2019 Jul 26;14(7):e0220492. doi: 10.1371/journal.pone.0220492 (PMC6660089; doi:10.1371/journal.pone.0220492)
Supplement: S1 Table — (DOCX) [file pone.0220492.s003.docx]

**S1 Table. List of *R. ruber* steroid mutants available**

| **Mutant** | **Genes deleted** | **Reference** |
| --- | --- | --- |
| *ΔkshA1* | *kshA1* | Laboratory collection |
| *ΔkshA2* | *kshA2* | Laboratory collection |
| *ΔkshA3* | *kshA3* | Laboratory collection |
| *ΔkshB* | *kshB* | Laboratory collection |
| *ΔkshA1, 2,3* | *kshA1, kshA2* and  *kshA3* | [9] |
| *ΔkshA1,2* | *kshA1* and *kshA2* | [9] |
| *ΔkshA1,3* | *kshA1* and *kshA3* | [9] |
| *ΔkshA2,3* | *kshA2* and *kshA3* | [9] |
| *ΔkshB-kshA1* | *kshB* and *kshA1* | [9] |
| *ΔkshB-kshA1-kstD1,2,3* | *kshB, kshA1, kstD1, kstD2* and *kstD3* | Laboratory collection |
| *ΔkshA1-kstD1,2,3* | *kshA1, kstD1, kstD2* and *kstD3* | Laboratory collection |
| *ΔkshB-kstD1,2,3* | *kshB, kstD1, kstD2*  and *kstD3* | This work |
| *ΔkstD1* | *kstD1* | [8] |
| *ΔkstD2* | *kstD2* | [8] |
| *ΔkstD3* | *kstD3* | [8] |
| *ΔkstD1,2,3* | *kstD1, kstD2* and *kstD3* | [8] |
| *ΔkstD1,2* | *kstD1* and *kstD2* | [8] |
| *ΔkstD1,3* | *kstD1*and *kstD3* | [8] |
| *ΔkstD2,3* | *kstD2* and *kstD3* | [8] |
| Δ*cho*G | *choG* | [7] |
| Δ*cho*G-*kstD*2 | *choG* and *kstD2* | Laboratory collection |

ChoG: cholesterol oxidase; KstD: 3-ketosteroid Δ^1^-dehydrogenase ; KshAB: 3-Ketosteroid 9α-Hydroxylase
