# Supplementary material for: Metabolic engineering of Rhodococcus ruber Chol-4: A cell factory for testosterone production
Source: PLoS One. 2019 Jul 26;14(7):e0220492. doi: 10.1371/journal.pone.0220492 (PMC6660089; doi:10.1371/journal.pone.0220492)
Supplement: S2 Table — (DOCX) [file pone.0220492.s004.docx]

**S2 Table. Microbial conversion of natural sterols to testosterone.**

| **Microorganism** | **Initial**  **Substrate** | **Supplemental**  **Carbon source (%)** | **TS as product (µM)** | **Conversion ratio %** | **Maximum accumulation of TS (h)** | **Reference** |
| --- | --- | --- | --- | --- | --- | --- |
| *Rhodococcus ruber strain Chol-4* | AD | 1% Glucose | 504 | 61 | 24 | This work |
| *Mycobacterium sp.* B-3805S | Phytosterols | 2% Glucose and 1% Peptone | 1190 | 31 | 120 | Lo CK, Pan CP, Liu WH. Production of testosterone from phytosterol using a single-step microbial transformation by a mutant of *Mycobacterium* sp. J Ind Microbiol Biotechnol. 2002;28(5):280-3. Epub 2002/05/03. doi: 10.1038/sj/jim/7000243. PubMed PMID: 11986932. |
| *Mycobacterium sp NRRL B-3805* | Cholesterol | 4% Glucose and 1% Peptone | 1340 | 54 | 120 | Liu WH, Lo CK. Production of testosterone from cholesterol using a single-step microbial transformation of *Mycobacterium* sp. J Ind Microbiol Biotechnol. 1997;19(4):269-72. Epub 1998/01/24. PubMed PMID: 9439002. |
| *Mycobacterium sp NRRL B-3686* | ADD | 5% Glycerol | 48.6 | nd | 72 | Hung B, Falero A, Llanes N, Pérez C, Ramirez MA. Testosterone as biotransformation product in steroid conversion by *Mycobacterium* sp. Biotechnology letters 1994;16(5):497-500. |
| *Mycobacterium sp.* Et1 | AD | 4% Glucose | 210 | 6 | 90 | Egorova OV, Nikolayeva VM, Sukhodolskaya GV, Donova M. Transformation of C 19-steroids and testosterone production by sterol-transforming strains of *Mycobacterium* spp. Journal of Molecular Catalysis B Enzymatic. 2009;57(1):198-203. doi: 10.1016/j.molcatb.2008.09.003. |
| *Mycobacterium smegmatis MS6039-5941* | Cholesterol | 1% Glucose (growing-cells)  1% Glycerol (pseudo-resting cells)l | 1000  900 | 75  68 | 69  69 | Fernández-Cabezón L, Galán B, García JL. Engineering *Mycobacterium smegmatis* for testosterone production. Microb Biotechnol. 2017;10(1):151-61. Epub 2016/11/20. doi: 10.1111/1751-7915.12433. PubMed PMID: 27860310; PubMed Central PMCID: PMCPMC5270716.. |
| *Lactobacillus bulgaricus* | AD | 4% Glucose | 1560 | nd | 96 | Kumar R, Dahiya JS, Singh D, Nigam P. Biotransformation of cholesterol using *Lactobacillus bulgaricus* in a glucose-controlled bioreactor. Bioresour Technol. 2001;78(2):209-11. PubMed PMID: 11333043. |

nd: not determined
